# Long- and short-term survival following laparoscopic and open pancreaticoduodenectomy for patients with periampullary tumors in Vietnam

**DOI:** 10.1016/j.amsu.2021.102690

**Published:** 2021-08-10

**Authors:** Tran Manh Hung, Tran Que Son, Tran Hieu Hoc, Tran Thanh Tung, Trieu Van Truong, Le Manh Cuong, Vu Duy Kien

**Affiliations:** a Department of General Surgery, Bach Mai Hospital, No. 78 Giai Phong Street, Hanoi, Viet Nam; b Hanoi Medical University, No. 1 Ton That Tung Street, Hanoi, Viet Nam; c National Hospital of Traditional Medicine, No. 29 Nguyen Binh Khiem Street, Hanoi, Viet Nam; d OnCare Medical Technology Company Limited, No. 77/508 Lang Street, Hanoi, Viet Nam

**Keywords:** Laparoscopic pancreaticoduodenectomy, Open pancreaticoduodenectomy, Cancer, Outcomes, Survival, Vietnam

## Abstract

**Background:**

Laparoscopic pancreaticoduodenectomy (LPD) is a less invasive alternative to the traditional open pancreaticoduodenectomy (OPD) approach used to treat periampullary tumors. However, previous studies examining the advantages of this surgery over OPD have produced mixed results. Here, a retrospective observational approach was used to compare the short- and long-term outcomes of patients with periampullary tumors who underwent LPD or OPD at a single institution in Vietnam.

**Materials and methods:**

Data were obtained from hospital medical records collected over five years from patients that underwent OPD or LPD. Information on demographics, medical status, tumor characteristics, operative variables, complications, and mortality was examined. Survival curves were constructed and the stepwise multivariate Cox proportional hazard model was used to identify the factors associated with the risk of death following surgery.

**Results:**

Eighty-four patients aged 26–80 years were included. Twenty-two patients underwent LPD and 62 received OPD. The operative time for the LPD group was significantly longer than that for the OPD group, and the LPD group was less likely to require a blood transfusion during surgery. While the short- and long-term survival rates did not differ for the procedures, the factors associated with the risk of death following surgery were tumors at the N1 stage and an age >65 years.

**Conclusion:**

Both LPD and OPD procedures for treating periampullary tumors exhibited comparable safety profiles, with similar short-term outcomes and long-term survival rates observed. Future studies with a larger sample size should be conducted to further examine the treatment outcomes following these surgical approaches.

## Highlights

• The OPD group was significantly more likely to require blood transfusion as compared to the LPD group (p=0.04).
• The operative time for the LPD group was significantly longer than that for the OPD group (p < 0.01).
• Factors associated with the risk of death following surgery were tumors at the N1 stage and age group > 65 years.

## 1 Introduction

Periampullary tumors include tumors in the area of the pancreatic head, distal bile duct, duodenum, and ampulla of Vater. Depending on the location of the tumor and the stage of the disease, patients with periampullary tumors often have a poor prognosis. Pancreaticoduodenectomy (PD) is the standard surgical method for thoroughly resolving tumors in the periampullary area [1]. However, PD is a challenging surgical procedure, as it involves the extensive dissection of visceral organs and reconstruction of the digestive tract [2,3]. Traditionally, open PD (OPD) was the main procedure used for patients with periampullary tumors. However, with the development of technology and improved surgical skills, laparoscopic PD (LPD) is now considered a minimally invasive alternative to OPD [[4], [5], [6]]. Some previous studies comparing LPD and OPD have shown no differences in efficacy and safety [[7], [8], [9], [10], [11]]. Nonetheless, LPD has some advantages for patients, including decreased intraoperative blood loss and a shortened hospital stay [7,9,[12], [13], [14], [15]]. While LPD was introduced in Vietnam in 2008, this procedure is mainly implemented in tertiary hospitals due to its complexity and technical difficulties. Although several previous studies have compared the LPD and OPD procedures in other countries [11,[13], [14], [15]], no studies have been conducted in Vietnam. Thus, this study aimed to review and compare the short-term and long-term outcomes of patients with periampullary tumors who underwent LPD or OPD at a single institution in Vietnam.

## 2 Methods

This was a retrospective observational study of patients with periampullary tumors treated at a tertiary hospital in Vietnam. The study was approved by Hanoi Medical University Institutional Ethical Review Board. The study registration identifying number (UIN) is researchregistry6970, which is available at https://www.researchregistry.com/. Data were obtained from hospital medical records collected over five years (2015–2020). The study has been reported in line with the STROCSS criteria [16]. All patients diagnosed with periampullary tumors and underwent OPD or LPD were included in the study. The periampullary tumors with pathologic confirmation included ampullary adenocarcinoma, pancreatic adenocarcinoma, cholangiocarcinoma, gastrointestinal stromal tumor (GIST), and intraductal pancreatic mucinous neoplasm (IPMN).

All patients had their medical histories recorded, and were given a clinical examination and laboratory tests in accordance with the hospital's guidelines. Patients were selected for the LPD or OPD procedure based on a decision by the hospital's consultation team. However, all patients were informed in detail about the advantages and disadvantages of LPD or OPD. All LPD and OPD procedures were performed by three experienced surgical teams. Each surgical team consisted of a main surgeon, two surgeon assistants, a nurse, an anesthesiologist and an anesthesiologist assistant. The OPD procedure was modified from the standard PD procedure with antrectomy or pylorus preservation, as described previously [1]. For the LPD procedure, the surgical team used five trocars (three 10 mm trocars and two 5 mm trocars) to perform the standard resection. The LPD techniques have been described in detail elsewhere [17].

The main outcome variables analyzed were mortality and the additional years lived after the operation. The patient variables included sex, age, Body Mass Index (BMI), comorbid disease (hypertension, diabetes, cardiac disease, pulmonary disease), laboratory test results (bilirubin, serum AST/ALT, albumin, creatine, urea, blood counts), and the American Society of Anesthesiologists (ASA) score. The operative variables included an estimation of blood loss, volume of blood transfusion, length of the operation, length of hospital stay, the occurrence of wound infection and several types of complications (bleeding, pancreatic fistula, gastrointestinal fistula, delayed gastric emptying and the Clavien-Dindo classification). The oncologic variables included tumor size, the type of tumor, the tumor stage, and the N stage. Pathologists at the hospital reviewed all specimens.

All continuous variables are presented as median and range, while the categorical variables are reported as frequency and percentage. Groups were compared using the Mann-Whitney *U* test for continuous variables, and the χ^2^ test or Fisher's exact test for categorical variables. Survival curves were drawn using the Kaplan-Meier method, and the difference between groups was compared using the log-rank test. The stepwise Cox regression model was used to identify factors associated with the risk of death following surgery. All statistical analyses were performed with STATA (version 14.0; Stata Corp, College Station, TX, USA), and a p-value < 0.05 was considered statistically significant.

## 3 Results

A total of 84 patients aged 26–80 years with periampullary tumors were included in the study. Of these patients, 22 underwent LPD and 62 received OPD. The general characteristics of the patients receiving each treatment are displayed in Table 1. Overall, the demographic and clinical characteristics of each group were similar. There was no sex difference between the LPD and OPD groups, but patient age in the OPD group was significantly higher than that in the LPD group (p < 0.05). There were no significant differences between the LPD and OPD groups in the incidence of non-communicable diseases, including hypertension, diabetes mellitus, cardiac disease, or pulmonary disease, nor were there differences in BMI or ASA. With the exception of albumin in the blood (p < 0.01), there were also no significant differences in the biochemical indices or complete blood count between the LPD and OPD groups.

**Table 1:** Characteristics of patients included in the study.

	Laparoscopic (n = 22)	Open (n = 62)	P value
	N (%) or median (range)		
Sex			
Men	12 (54.6)	35 (56.5)	0.88^†^
Women	10 (45.4)	27 (43.6)	
Age Group (year)			
20-39	3 (13.6)	1 (1.6)	0.06^†^
40-59	11 (50.0)	29 (46.8)	
60 and older	8 (36.4)	32 (51.6)	
BMI group			
<25	21 (95.5)	59 (95.2)	1.0^¢^
≥25	1 (4.5)	2 (4.8)	
Hypertension	2 (9.1)	7 (11.3)	1.0^¢^
Diabetes Mellitus	6 (27.3)	16 (25.8)	0.89^†^
Cardiac disease	1 (4.5)	7 (11.3)	0.67^¢^
Pulmonary disease	3 (13.6)	6 (9.7)	0.69^¢^
ASA			
1	4 (18.2)	7 (11.3)	0.56^¢^
2	13 (59.1)	44 (71.0)	
3	5 (22.7)	11 (17.7)	
Total bilirubin (μmol/L)	88.9 (4.1–248.7)	150.4 (4.0–498.5)	0.42^‡^
Serum AST (U/L)	74.5 (13.0–284.0)	66.0 (18–520.0)	0.54^‡^
Serum ALT (U/L)	80.0 (12.0–394.0)	71.5 (12.0–590.0)	0.81^‡^
Albumin (g/L)	38.9 (28.4–45.7)	34.1 (23.5–45.3)	<0.01^‡^
Creatinin (μmol/L)	69.5 (49.0–105.0)	72.0 (37.0–114.0)	0.90^‡^
Urea (mmol/L)	5.1 (1.8–7.8)	4.5 (1.4–11.4)	0.26^‡^
Red blood cell (10^12^/L)	4.2 (3.5–5.7)	4.1 (2.8–5.3)	0.45^‡^
White blood cell (10^9^/L)	7.5 (4.0–16.2)	8.1 (3.7–21.5)	0.40^‡^
Platelet (10^9^/L)	315.0 (163.0–642.0)	324.5 (124.0–574.5)	0.64^‡^

^†^χ^2^ test.

^‡^Mann-Whitney *U* test; ^¢^: Fisher's exact test.

Table 2 shows the operative details and complications for patients undergoing LPD or OPD. There was not a significant difference in blood loss between the two groups, but the OPD group was significantly more likely to require blood transfusion as compared to the LPD group (p = 0.04). In addition, the operative time for the LPD group was significantly longer than that for the OPD group (p < 0.01). However, there was no difference in the length of hospital stay between the two groups. Although the occurrence of wound infection in the OPD group (12.9%) was higher than that in the LPD group (0.0%), this difference did not reach statistical significance (p > 0.05). There were also no significant differences between the two groups in the rate of post-surgical complications, including bleeding, pancreatic fistula, gastrointestinal fistula, intestinal obstruction, and delayed gastric emptying, or in the Clavien-Dindo classification.

**Table 2:** Operative details and complications for patients with periampullary tumors receiving laparoscopic or open pancreaticoduodenectomy.

	Laparoscopic (n = 22)	Open (n = 62)	P value
	N (%) or median (range)		
Blood loss (mL)	337.0 (150.0–850.0)	283.5 (150.0–1340.0)	0.61^‡^
Blood transfusion (mL)	0.0 (0.0–700.0)	0.0 (0.0–2100.0)	0.04^‡^
Operative time (hour)	4.9 (3.0–6.0)	3.9 (3.0–5.6)	<0.01^‡^
Hospital stays (day)	13.5 (9.0–30.0)	12.0 (8.0–31.0)	0.27^‡^
Wound infection	0 (0.0)	8 (12.9)	0.10^¢^
Bleeding			
Grade A	0 (0.0)	1 (1.6)	0.60^¢^
Grade B	0 (0.0)	1 (1.6)	
Grade C	1 (4.6)	0 (0.0)	
Pancreatic fistula			
Grade A	1 (4.6)	1 (1.6)	0.47^¢^
Grade B	2 (9.1)	4 (4.5)	
Grade C	1 (4.6)	1 (1.6)	
Gastrointestinal fistula	1 (4.6)	1 (1.6)	0.46^¢^
Intestinal obstruction	0 (0.0)	2 (3.2)	1.00^¢^
Delayed gastric emptying			
Grade A	1 (4.6)	3 (4.8)	0.24^¢^
Grade B	1 (4.6)	1 (1.6)	
Grade C	1 (4.6)	1 (1.6)	
Surgical complication (Clavien-Dindo)			
Grade I	1 (4.6)	3 (4.8)	0.11^¢^
Grade II	3 (13.6)	8 (12.9)	
Grade IIIa	1 (4.6)	3 (4.8)	
Grade IIIb	2 (9.1)	0 (0.0)	
Grade IVa	1 (4.6)	0 (0.0)	

^‡^: Mann-Whitney *U* test; ^¢^: Fisher's exact test.

The oncologic variables for both patient groups are shown in Table 3. With the exception of tumor size, which was significantly larger in the OPD group (p < 0.01), the other variables did not significantly differ.

**Table 3:** Oncologic variables for patients with periampullary tumors receiving laparoscopic and open pancreaticoduodenectomy.

	Laparoscopic (n = 22)	Open (n = 62)	P value
	N (%) or median (range)		
Tumor size (mm)	19.5 (9.0–30.0)	28.0 (10.0–78.0)	<0.01^‡^
Type of tumor			
Ampullary adenocarcinoma	16 (72.8)	28 (45.2)	0.28^¢^
Pancreatic adenocarcinoma	3 (13.6)	17 (27.4)	
Cholangiocarcinoma	3 (13.6)	13 (21.0)	
Gastrointestinal stromal tumor (GIST)	0 (0.0)	1 (1.6)	
Intraductal pancreatic mucinous neoplasm (IPMN)	0 (0.0)	3 (4.8)	
Tumor stage			
T1	1 (4.6)	1 (1.6)	0.21^¢^
T2	5 (22.8)	28 (45.2)	
T3	15 (68.2)	30 (48.4)	
T4	1 (4.6)	3 (4.8)	
N stage			
N0	7 (31.8)	13 (21.0)	0.38^¢^
N1	15 (68.2)	49 (79.0)	

‡: *t*-test; ^¢^: Fisher's exact test.

**Fig. 1:**
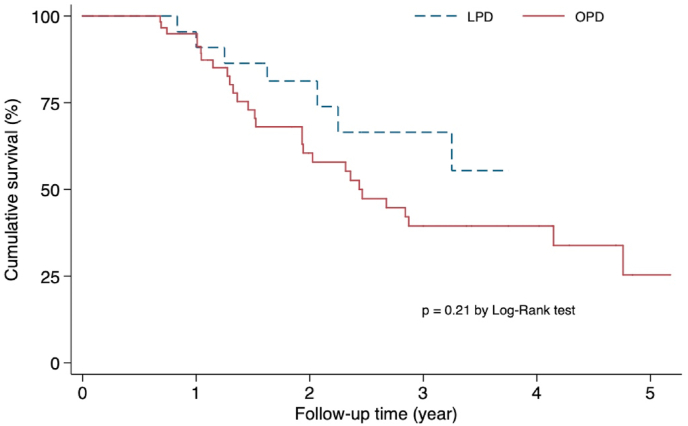
Kaplan-Meier estimated survival curves for 84 patients with periampullary tumors undergoing laparoscopic or open pancreaticoduodenectomy. LPD: laparoscopic pancreaticoduodenectomy; OPD: open pancreaticoduodenectomy.

The survival curves for both procedures are shown in Fig. 1. The 1-year survival rate was quite similar in the LPD (90.9%) and OPD (94.9%) groups. The 3-year survival rate in the LPD group (66.5%) was considerably higher than that in the OPD group (39.5%). However, the log-rank test revealed no significant difference in survival rates over time (p > 0.05). The stepwise multivariate Cox proportional hazard model indicated that the significant factors associated with the risk of death following surgery were tumors at the N1 stage (HR = 2.73, 95% CI = 1.06–7.02; p = 0.04), age group > 65 years (HR = 3.77, 95% CI = 1.57–9.05; p < 0.01) (Table 4).

**Table 4:** Stepwise multivariate Cox proportional hazard model showing the effect of factors on the risk of death for 84 patients with periampullary tumors.

Variable	HR (95% CI)	P value
N stage (N1 vs. N0)	2.73 (1.06–7.02)	0.04
Sex (Men vs. Women)	0.57 (0.28–1.14)	0.16
Age group (>65 years vs. ≤ 65 years)	3.77 (1.57–9.05)	<0.01
Diabetes Mellitus (Yes vs. No)	0.54 (0.23–1.27)	0.19
Operation (Laparoscopic vs. Open)	0.53 (0.22–1.27)	0.15

List of abbreviations: HR: Hazard Ratio; CI: Confidence Interval.

## 4 Discussion

This is the first study in Vietnam to compare the short- and long-term outcomes following LPD and OPD for patients with periampullary tumors. We had hoped that this study would help hospitals invest more resources to improve LPD procedures. As the PD procedure is a difficult and complicated operation [1], the option of a minimally invasive approach, such as LPD, can help reduce the risk of infection and the length of hospital stay [4,7,11,15]. Previous research had also indicated that the LPD procedure could increase a patient's quality of life [18]. The current study found no differences in the short- and long-term survival outcomes between LPD and OPD groups, which is consistent with some previous work [11,13,15,19]. However, several factors related to the long-term survival of patients were identified and should be considered to improve the future treatment of periampullary tumors.

In the current study, the survival rate of patients receiving LPD tended to be higher than that of patients receiving OPD; however, similar to previous work [11,13,15,19], this difference was not statistically significant. The current results are also in line with other studies showing that patients receiving LPD are less likely to need a blood transfusion compared with those receiving OPD [11,20]. Previous work has also demonstrated that the LPD procedure is associated with decreased blood loss, wound infection, intensive care admission and hospital stay, compared to OPD [10,11,[21], [22], [23]]. However, these differences were not observed here. While the reasons for this discrepancy are unclear, they may relate to sample size variation and how the variables were measured in the previous work. In addition, it is likely that an improvement in the surgeon's skill over time increased control over the operation [20,[24], [25], [26]], thus resulting in no differences in blood loss and hospital stay between the LPD and OPD groups. Felix et al., in a systematic review of three randomized controlled trials, also showed no difference between the LPD and OPD procedures in short-term outcomes, including 90-day mortality, postoperative complications, blood loss, and length of hospital stay [23,[27], [28], [29]].

Similar to other studies, we also found no differences in the distribution of stage of cancer between the LPD and OPD groups [11,15,19,20]. In a previous systematic review, it was also mentioned that no differences related to the stage of cancer were reported for LPD and OPD groups in earlier research [10,22]. However, we did observe that patients at the N1 stage had an increased the risk of death compared to those at N0, an issue that was not noted in previous work [11,15,19]. This observation is consistent with clinical practice, as patients at the N1 stage have a more severe condition than those at the N0 stage. In addition, we also found an increased risk of death for patients 65 years old and older as compared to younger patients. Similar results were reported by Olga et al., who also observed that increased age was associated with a higher risk of death in patients with periampullary tumors [19]. In contrast to these results, John et al. and Kristopher et al. did not find any differences related to the risk of death between patients 65 years old and older and younger patients [11,13]. Future studies examining age differences in the risk of death among patients with periampullary tumors may need to include a larger sample size.

Several postoperative complications were observed in the current study, but no differences in the rate of complications occurred across the LPD and OPD groups. As some postoperative complications can delay the timing of subsequent chemotherapy, the emergence of surgical complications can also indirectly affect the patient's long-term survival [13,19]. A previous study reported that the LPD procedure helped patients access adjuvant therapy earlier than the OPD procedure [15]. Although we intended to examine the time to access adjuvant therapy in the current study, the data in the medical records was inadequate and thus could not be analyzed. Usually, patients receiving PD would be transferred to the department of oncology to continue with chemotherapy 6–8 weeks following the surgery, and would receive a standard chemotherapy treatment protocol. We believe that, in addition to studying the effects of the PD procedure, additional examinations of the follow-up treatment phases will be necessary to improve patient's survival outcomes.

We performed both LPD and OPD procedures in our hospital simultaneously. The number of patients included in this study comprised all patients who underwent LPD (from June 2016 to December 2020) or OPD procedures (from January 2015 to December 2020) in our facility. Furthermore, as our hospital is a tertiary hospital, most patients with periampullary tumors in this region were treated here. We found no differences between patients receiving LPD or OPD regarding sex group, age, BMI or chronic comorbid diseases. Furthermore, the laboratory results and tumor characteristics of patients before surgery were relatively similar. Therefore, patients in the LPD and OPD groups were comparable, ensuring that any differences observed in treatment outcomes were not attributable to these factors.

It is important to note that the current study has several limitations. First, a retrospective approach was used; thus, selection bias may have potentially impacted the interpretation of the results. In addition, the sample size was relatively small and data were collected from a single center. Thus, generalization to other settings or the general population may be limited. Furthermore, several other factors that may have been relevant to the study results were not examined. For example, separation of the groups by tumor type or tumor stage may have yielded differing results. It should also be noted that patients with smaller tumor sizes were more likely to be selected for the LPD procedure, which may have affected the comparison between the LPD and OPD procedures.

## 5 Conclusion

Here, we found that both LPD and OPD procedures for treating periampullary tumors were performed safely, with similar short-term outcomes and long-term survival rates observed. However, patients treated with LPD were less likely to get blood transfusions during surgery and experienced a longer operative duration. The factors associated with an increased risk of death following surgery included tumors at the N1 stage and an age greater than 65. Futures studies aimed at examining the short- and long-term outcomes following LPD and OPD procedures, and identifying the factors related to long-term survival, would benefit from a large sample size study and a prospective approach.

## Ethical approval

The study was approved by Hanoi Medical University Institutional Ethical Review Board (decision no. 04/HDDDDHYHN, dated 06/01/2017).

## Sources of funding

No funding was received in this study.

## Author contribution

TMH, TQS, VDK contributed to the conception and design of the study, data collection, analyzed and interpreted the data, drafted the manuscript. THH, TVT, TTT, LMC reviewed literature, interpreted the data, provided critical input to the manuscript. All authors read and approved the final manuscript.

## Registration of research studies

1. Name of the registry: researchregistry.com
2. Unique Identifying number or registration ID: researchregistry6970
3. Hyperlink to your specific registration (must be publicly accessible and will be checked): https://www.researchregistry.com/register-now#home/registrationdetails/60ef2c339fc52b001effd3a1/

## Consent

Need for written informed consent was waived owing to the retrospective nature of the study.

## Guarantor

Tran Manh Hung, M.D., Ph. D.

## Availability of data and materials

The datasets used and/or analyzed during the current study are available from the corresponding author on reasonable request.

## Consent for publication

Not applicable.

## Provenance and peer review

Not commissioned, externally peer-reviewed.

## Declaration of competing interest

We have nothing to declare.
